# PGC-1α Controls Mitochondrial Biogenesis in Drug-Resistant Colorectal Cancer Cells by Regulating Endoplasmic Reticulum Stress

**DOI:** 10.3390/ijms20071707

**Published:** 2019-04-05

**Authors:** Chul Won Yun, Yong-Seok Han, Sang Hun Lee

**Affiliations:** 1 Medical Science Research Institute, Soonchunhyang University Seoul Hospital, Seoul 336-745, Korea; skydbs113@naver.com (C.W.Y); format7000@naver.com (Y.-S.H); 2 Departments of Biochemistry, Soonchunhyang University College of Medicine, Cheonan 330-930, Korea

**Keywords:** colorectal cancer cells, PGC-1α, mitochondrial biogenesis, drug resistance, endoplasmic reticulum stress, apoptosis

## Abstract

Anti-cancer drug resistance is a serious issue for patients with colorectal cancer (CRC). Although recent studies have shown the mechanism by which CRC cells become drug resistant, novel strategies for overcoming this drug resistance have not yet been developed. To address this problem, we characterized 5-fluorouracil (5FU)-resistant CRC cells after treatment with 5FU, and focused on the expression of peroxisome proliferator-activated receptor gamma coactivator 1-alpha (PGC-1α) in these cells. In 5FU-resistant CRC cells, the 5FU did not considerably decrease the mitochondrial biogenesis or mitochondrial complex I and IV activities, and only partially decreased the antioxidant enzymatic activity, oxygen consumption ratio, and cell survival. The expression of PGC-1α was remarkably increased in the 5FU-resistant CRC cells compared with the 5FU-sensitive CRC cells. The 5FU-resistant CRC cells displayed enhanced mitochondrial biogenesis, oxidative phosphorylation, and antioxidant enzyme activities against 5FU-induced reactive oxygen species, because of the increased expression of PGC-1α. PGC-1α inhibited 5FU-induced endoplasmic reticulum (ER) stress in the 5FU-resistant CRC cells, resulting in the suppression of apoptosis. These findings reveal that PGC-1α plays an important role in drug resistance in 5FU-resistant CRC cells. Moreover, PGC-1α could serve as a novel target in patients with 5FU-resistant CRC.

## 1. Introduction

Colorectal cancer (CRC) is the third most common health problem worldwide, and the fourth leading cause of mortality [1]. Generally, treatments for CRC include surgery, chemotherapy, and radiotherapy [2]. 5-fluorouracil (5FU) is a fluoropyrimidine analogue and is used to treat several cancers, such as colorectal [3], breast [4], and gastric cancers [5]. 5FU acts as an anticancer agent by inhibiting thymidylate synthase activity, DNA synthesis, and DNA repair in cancer cells, resulting in cell death [6,7]. However, drug resistance leads to cancer treatment failure and an increased likelihood of cancer recurrence and metastasis [8]. Indeed, the low therapeutic efficacy of chemotherapy as a result of drug resistance causes most cancer-related deaths. The decrease in drug efficacy usually occurs because of alterations in cancer cell physiology, such as the mutation of the target protein, damage to DNA repair, increased anticancer drug efflux, and the activation of alternative survival signaling pathways [9,10]. Therefore, understanding the mechanisms related to drug resistance is important when developing novel therapeutic strategies. 

Mitochondria are important organelles for energy metabolism, apoptosis, and adaptation to stressful conditions in mammalian cells [11]. Mitochondria play an important role in cancer metabolic reprogramming, given their function in catabolic and anabolic processes such as adenosine triphosphate (ATP) production, mitochondria respiration, and the biosynthesis of macromolecules [12,13]. In addition, mitochondria are associated with drug resistance in cancer cells. A recent study revealed that metabolic modulation is related to cancer cell differentiation, proliferation, and apoptosis, suggesting that metabolic enzymes may be involved in drug resistance in cancer cells [14]. Therefore, understanding the mechanisms associated with mitochondrial function in cancer may be an essential factor to developing key novel therapeutic strategies.

Peroxisome proliferator-activated receptor gamma coactivator 1-alpha (PGC-1α) is a transcriptional coactivator that interacts with peroxisome proliferator-activated receptor gamma [15,16]. PGC-1α is highly expressed in the mitochondria and tissues in response to the high demand for energy related to energy metabolism, mitochondrial biogenesis, homeostasis, and other biological pathways [17,18,19]. Recent studies have clarified the expression levels of PGC-1α related to cancer progression, proliferation, invasion, and metabolic pathways via the modulation of the mitochondrial function in diverse cancers [20,21,22]. In addition, it has been reported that PGC-1α acts as a key regulator of the mitochondrial metabolism by inducing a bioenergetic potential and promoting the acquisition of drug resistance acquisition by metabolic adaptation [23]. Knowledge of the mechanisms of PGC-1α is required in order to understand drug resistance related to the regulation of mitochondria in cancer.

Despite several studies on drug resistance, the mechanism by which 5FU produces chemoresistant CRC cells remains unclear. In this study, to reveal the underlying mechanism responsible for 5FU-resistant CRC cells, we assessed the effects of 5FU on mitochondrial biogenesis in CRC cells. Furthermore, we investigated the role of PGC-1α on the mitochondrial function and mitochondria-mediated apoptosis following anticancer drug-induced endoplasmic reticulum (ER) stress.

## 2. Results

### 2.1. Expression of PGC-1α and Mitochondrial Function in 5FU-Resistant CRC Cells

**Figure 1:**
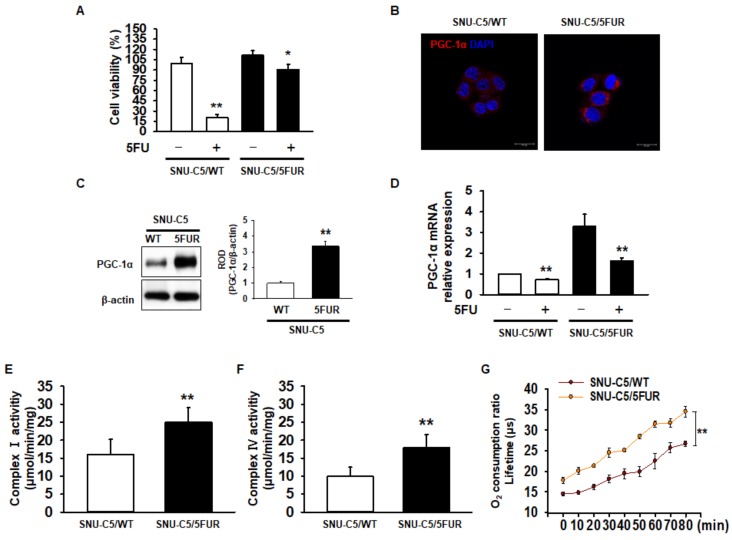
Assessment of mitochondrial function in 5-fluorouracil (5FU)-resistant colorectal cancer (CRC) cells. (**A**) Cell viability in 5FU-sensitive SNU-C5 (SNU-C5/WT) and 5FU-resistant SNU-C5 (SNU-C5/5FUR) cells after treatment with 5FU (140 μM) for 24 h (*n* = 3; biological replicates). (**B**) The expression of peroxisome proliferator-activated receptor gamma coactivator 1-alpha (PGC-1α) (red) in the SNU-C5/WT and SNU-C5/5FUR cells was analyzed by immunocytochemistry. The nuclei were stained by 4′,6-diamidino-2-phenylindole (DAPI) (blue). Scale bar = 100 μm (*n* = 3; biological replicates). (**C**) The expression of PGC-1α in the SNU-C5/WT and SNU-C5/5FUR cells treated with 5FU (140 μM) for 24 h was analyzed by Western blot (*n* = 3; biological replicates). (**D**) The mRNA expression of PGC-1α in the SNU-C5/WT and SNU-C5/5FUR cells with or without 5FU treatment. (**E**,**F**) The mitochondrial complex I (**E**) and IV (**F**) activity was measured in the SNU-C5/WT and SNU-C5/5FUR cells treated with 5FU (140 μM) for 24 h (*n* = 3; biological replicates). (**G**) Oxygen consumption ratio in the SNU-C5/WT and SNU-C5/5FUR cells after treatment with 5FU (140 μM) (*n* = 3; biological replicates). Values represent means ± standard error of the mean (SEM). * *p* < 0.05 vs. the control; ** *p* < 0.01 vs. the control.

To assess drug resistance in CRC cells, we analyzed cell survival in the 5FU-sensitive CRC cell line SNU-C5 (SNU-C5/WT), and 5FU-resistant SNU-C5 (SNU-C5/5FUR) cells after treatment with 5FU. Our cell viability assay indicated that 5FU treatment significantly decreased the cell survival (Figure 1A). However, an increased reduction in the cell survival ratio was higher in the SNU-C5/WT cells than in the SNU-C5/5FUR cells (Figure 1A). Recent studies have suggested that PGC-1α enhances the mitochondrial energy metabolism and biogenesis in melanoma, colorectal cancer, and endometrial carcinoma [24,25,26]. Moreover, PGC-1α is related to the drug resistance that occurs in ovarian cancer cells [27]. Given these facts, we sought to determine the expression of PGC-1α in SNU-C5/WT and SNU-C5/5FUR cells via immunocytochemistry and Western blot analyses. The expression of PGC-1α was significantly increased in the SNU-C5/5FUR cells, compared with that in the SNU-C5/WT cells (Figure 1B,C), which indicates that the expression of PGC-1α is increased in the 5FU-resistant CRC cells. Also, we confirmed that the mRNA expression of PGC-1α decreased after 5FUR treatment compared with the SNU-C5/WT cells, and that PGC-1α was highly expressed in the absence or presence of 5FU in the SNU-C5/5FUR cells compared with the SNU-C5/WT cells (Figure 1D). To further investigate the mitochondrial function within the SNU-C5/WT and SNU-C5/5FUR cells, the activities of mitochondrial complex I and IV and of the oxygen consumption ratio were analyzed. The activities of mitochondrial complex I and IV and of the oxygen consumption ratio were significantly increased in the SNU-C5/5FUR cells (Figure 1E,F) compared with that in the SNU-C5/WT cells (Figure 1G). These data indicated that the PGC-1α expression and mitochondrial function increased in the 5FU-resistant CRC cells.

### 2.2. PGC-1α Regulates the Mitochondrial Function in 5FU-Resistant CRC Cells

**Figure 2:**
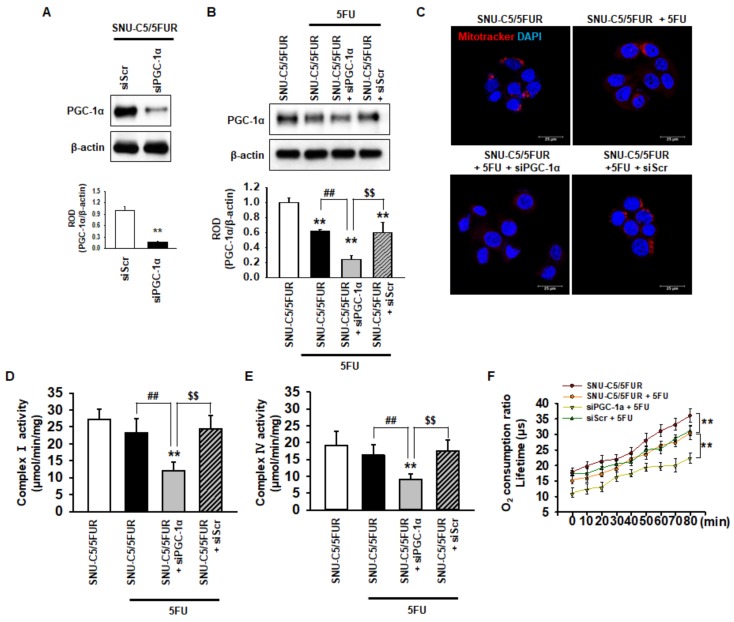
PGC-1α regulates mitochondrial function in 5FU-resistant CRC cells. (**A**) Expression of PGC-1α after transfection of the SNU-C5/5FUR cells with PGC-1α siRNA (siPGC-1α) (*n* = 3; biological replicates). (**B**) The expression level of PGC-1α in the siPGC-1α-transfected SNU-C5/5FUR cells after treatment with 5FU (140 μM) for 24 h (*n* = 3; biological replicates). (**C**) SNU-C5/5FUR cells treated with 5FU (140 μM) for 24 h after transfection with siPGC-1α and siScramble (siScr). The morphology of the mitochondria was analyzed by Mitotracker (Red) staining. The nuclei were stained by DAPI (blue). Scale bar = 20 μm (*n* = 3; biological replicates). (**D**,**E**) The mitochondrial complex I (**D**) and IV (**E**) activity was measured in siPGC-1α-transfected SNU-C5/5FUR in the presence of 5FU (140 μM) for 24 h (*n* = 3; biological replicates). Values represent the means ± SEM. ** *p* < 0.01 vs. untreated SNU-C5/5FUR; ## *p* < 0.01 vs. SNU-C5/5FUR after treatment with 5FU; $$ *p* < 0.01 vs. SNU-C5/5FUR+siPGC-1α after treatment with 5FU. (**F**) The oxygen consumption ratio in the siPGC-1α-transfected SNU-C5/5FUR cells after treatment with 5FU (140 μM) for 24 h (*n* = 3; biological replicates). Values represent the means ± SEM of triplicate experiments. ** *p* < 0.01.

PGC-1α is associated with mitochondrial biogenesis and functionality [28]. To assess the effect of PGC-1α on the mitochondria in 5FU-resistant CRC cells, we knocked down the expression of PGC-1α in SNU-C5/5FUR cells (Figure 2A). After treatment of the SNU-C5/5FUR cells with 5FU, we analyzed the expression of PGC-1α, the mitochondrial morphology, the mitochondrial complex I and IV activities, and the oxygen consumption ratio. In the SNU-C5/5FUR cells treated with 5FU, the expression of PGC-1α was increased and the knockdown of PGC-1α inhibited the 5FU-induced increase of PGC-1α (Figure 2B). Treatment with 5FU did not significantly alter the mitochondrial morphology (Figure 2C). In addition, our mitochondrial functional assays (i.e., complex I and IV activity assay and the analysis of the oxygen consumption ratio) have shown that 5FU did not change the activities of mitochondrial complex I and IV in the SNU-C5/5FUR cells, although the oxygen consumption ratio was significantly decreased after the treatment of SNU-C5/5FUR cells with 5FU (Figure 2D–F). Transfection with siPGC-1α alone slightly decreased mitochondrial complex I and IV activity in the SNU-C5/5FUR cells (Supplemental Figure S1). However, the silencing of PGC-1α significantly decreased the mitochondrial mass, the activities of mitochondrial complex I and IV, and the oxygen consumption ratio in the SNU-C5/5FUR cells after treatment with 5FU (Figure 2C–F), indicating that PGC-1α is involved in the mitochondrial functionality in the 5FU-resistant CRC cells against treatment with 5FU.

### 2.3. PGC-1α Inhibits Generation of Reactive Oxygen Species (ROS) in 5FU-Resistant CRC Cells Through an Increase in Antioxidant Enzyme Activities

**Figure 3:**
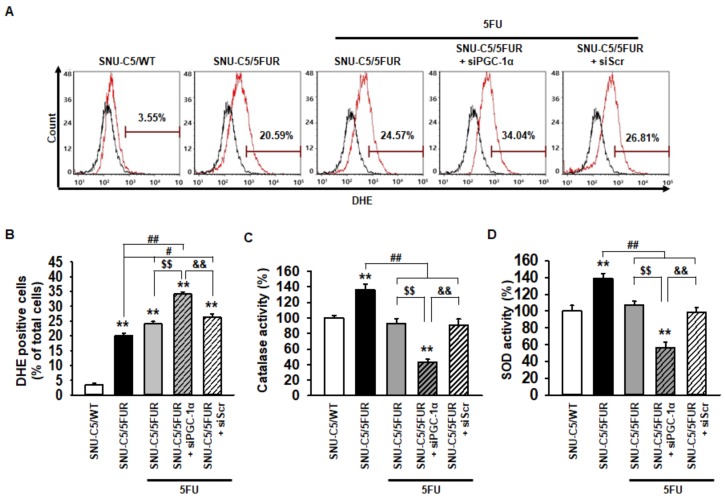
Effect of PGC-1α on the antioxidant enzyme activity in SNU-C5/5FUR cells. (**A**) SNU-C5/5FUR cells treated with 5FU (140 μM) for 24 h, following transfection with siPGC-1α and siScramble. The reactive oxygen species (ROS) were detected by dihydroethidium (DHE) staining using flow cytometry (black lines denotes the negative control, red lines indicate DHE staining for each group) (*n* = 3; biological replicates). (**B**) Bar graph indicating the quantification of DHE-positive cells. (**C** and **D**) Activities of the catalase (**C**) and superoxide dismutase (SOD) (**D**) were measured by activity assay (*n* = 3; biological replicates). Values represent the means ± SEM. ** *p* < 0.01 vs. SNU-C5/WT; # *p* < 0.05; ## *p* < 0.01 vs. Unntreated SNU-C5/5FUR; $$ *p* < 0.01 vs. SNU-C5/5FUR treated with 5FU; && *p* < 0.01 vs. SNU-C5/5FUR+siPGC-1α treated with 5FU.

To explore whether PGC-1α regulates the production of ROS in 5FU-resistant CRC cells, flow cytometry analysis for dihydroethidium (DHE) staining was performed in the SNU-C5/5FUR cells after treatment with 5FU. Although treatment with 5FU significantly increased the ROS production in the SNU-C5/5FUR cells, the generation of ROS was more significantly increased in the PGC-1α-silenced SNU-C5/5FUR cells than in the control SNU-C5/5FUR cells after treatment with 5FU (Figure 3A,B). In addition, after 5FU exposure, the catalase and superoxide dismutase (SOD) activities were significantly decreased in the PGC-1α-silenced SNU-C5/5FUR cells compared with those in the control SNU-C5/5FUR cells (Figure 3C,D). No effects of catalase and SOD activity were evident in the siPGC-1α transfected group (Supplemental Figure S2). These results demonstrated that PGC-1α suppresses ROS production in the 5FU-resistant CRC cells via the induction of antioxidant enzyme activities, and thus lessens the efficacy of 5FU treatment on CRC cells.

### 2.4. PGC-1α Protects from 5FU-Induced Endoplasmic Reticulum (ER) Stress in 5FU-Resistant CRC Cells

**Figure 4:**
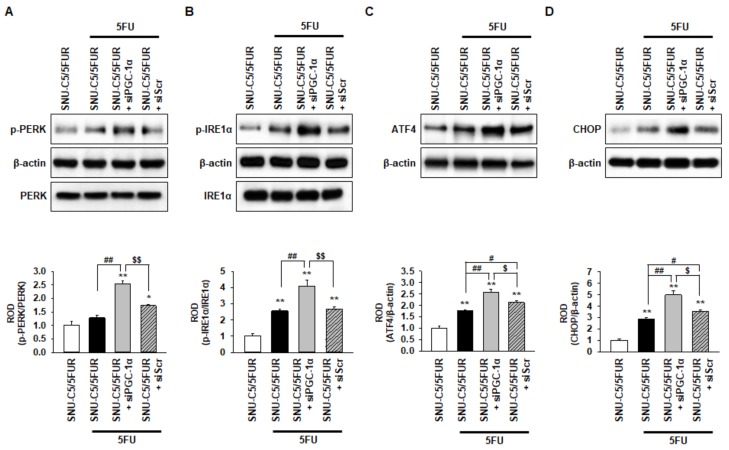
Protective effect of PGC-1α in SNU-C5/5FUR cells against 5FU-induced endoplasmic reticulum (ER) stress. (**A**–**D**) The expression level of ER stress markers, phospho-protein kinase-like endoplasmic reticulum kinase (p-PERK) (**A**), phospho-inositol-requiring enzyme 1 alpha (p-IRE1α) (**B**), activating transcription factor 4 (ATF4) (**C**), and C/EBP homologous protein (CHOP) (**D**) in siPGC-1α-transfected SNU-C5/5FUR cells after treatment with 5FU (140 μM) for 24 h (*n* = 3; biological replicates). The bar graphs represent the quantification of the ER stress marker expression as determined by the densitometry relative to PERK, IRE1α, and β-actin. Values represent the means ± SEM. ** *p* < 0.01 vs. SNU-C5/WT; # *p* < 0.05; ## *p* < 0.01 vs. SNU-C5/5FUR treated with 5FU; $ *p* < 0.05; $$ *p* < 0.01 vs. SNU-C5/5FUR+siPGC-1α treated with 5FU.

A previous study has shown that 5FU induces ER stress, resulting in apoptotic cell death in hepatocellular carcinoma [29]. To reveal whether 5FU induces ER stress in 5FU-resistant CRC cells, and to determine if PGC-1α regulates ER stress following treatment with 5FU, we assessed the activation of the ER stress markers, including protein kinase-like endoplasmic reticulum kinase (PERK), inositol-requiring enzyme 1 alpha (IRE1α), activating transcription factor 4 (ATF4), and C/EBP homologous protein (CHOP), in the SNU-C5/5FUR cells after treatment with 5FU. The level of p-PERK was not significantly changed in the SNU-C5/5FUR cells treated with 5FU compared with the untreated SNU-C5/5FUR cells (Figure 4A). However, the activation of IRE1α and the expression of ATF4 and CHOP were significantly increased in the SNU-C5/5FUR cells treated with 5FU, compared with the untreated SNU-C5/5FUR cells (Figure 4B–D), indicating that 5FU partially induces ER stress in the 5FU-resistant cells. Interestingly, silencing the PGC-1α drastically increased the levels of p-PERK, p-IRE1α, ATF4, and CHOP in the SNU-C5/5FUR cells treated with 5FU (Figure 4A–D). There was no effect of an ER stress marker expression in the siPGC-1α transfected group (Supplemental Figure S3). These findings suggest that PGC-1α is a key regulator of ER stress in th 5FU-resistant CRC cells in the presence of 5FU.

### 2.5. PGC-1α Inhibits 5FU-Induced Apoptosis in 5FU-Resistant CRC Cells

**Figure 5:**
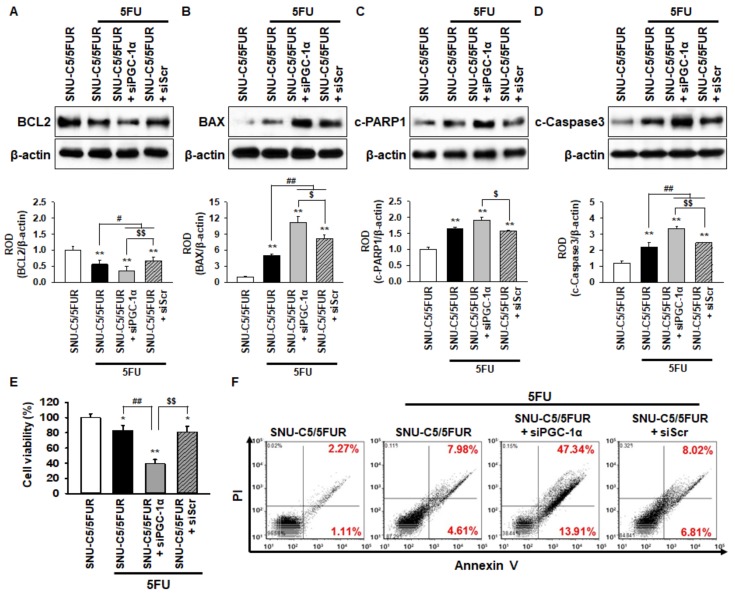
PGC-1α protects against SNU-C5/5FUR apoptosis caused by 5FU-induced ER stress. (**A**–**D**) The levels of apoptosis-related proteins, including anti-apoptotic protein B-cell lymphoma 2 (BCL2) (**A**), pro-apoptotic proteins Bcl-2-associated X protein (BAX) (**B**), cleaved caspase 3 (c-Caspase 3; **C**), and apoptotic protein cleaved poly (ADP-ribose) polymerase 1 (c-PARP1; **D**) in SNU-C5/5FUR cells treated with siPGC-1α after treatment with 5FU (140 μM) for 24 h (*n* = 3; biological replicates). The bar graphs represent the quantification of the expression levels as determined by densitometry relative to β-actin. (**E**) The cell viability of the SNU-C5/5FUR cells treated with siPGC-1α after treatment with 5FU (140 μM) for 24 h (*n* = 3; biological replicates). (**F**) Flow cytometry for propidium iodide (PI)/annexin V staining in the SNU-C5/5FUR cells treated with siPGC-1α after treatment with 5FU (140 μM) for 24 h (*n* = 3; biological replicates). Values represent the means ± SEM. * *p* < 0.05; ** *p* < 0.01 vs. SNU-C5/WT; # *p* < 0.05; ## *p* < 0.01 vs. SNU-C5/5FUR treated with 5FU; $ *p* < 0.05; $$ *p* < 0.01 vs. SNU-C5/5FUR+siPGC-1α treated with 5FU.

To examine whether PGC-1α protects against apoptosis in the 5FU-resistant CRC cells when treated with 5FU, the expression of apoptosis-related proteins in the SNU-C5/5FUR cells was assessed by Western blot after treatment with 5FU. The level of anti-apoptotic protein B-cell lymphoma 2 (BCL2) in the SNU-C5/5FUR cells treated with 5FU was significantly decreased compared with that in the untreated SNU-C5/5FUR cells (Figure 5A). The expression of pro-apoptotic proteins, such as Bcl-2-associated X protein (BAX), cleaved caspase-3, and apoptotic protein, as well as cleaved Poly (ADP-ribose) polymerase 1 (c-PARP1), were significantly increased in the 5FU-treated SNU-C5/5FUR cells compared with the untreated SNU-C5/5FUR cells (Figure 5B–D). However, in the presence of 5FU, silencing PGC-1α in the SNU-C5/5FUR cells significantly decreased the anti-apoptotic protein and significantly increased the pro-apoptotic proteins (Figure 5A–D). In addition, the cell viability and flow cytometry analysis for propidium iodide (PI)/annexin V have shown that the knockdown of PGC-1α significantly induced the apoptosis of SNU-C5/5FUR cells after treatment with 5FU (Figure 5E and 5F). No effects of apoptosis-related markers expression and cell viability were evident in the siPGC-1α transfected group (Supplemental Figure S4). These data demonstrate that PGC-1α is involved in 5FU-induced apoptosis in drug-resistant CRC cells.

## 3. Discussion

In this study, we revealed that the level of PGC-1α is increased in 5FU-resistant CRC cells, and PGC-1α enhanced cancer cell survival when exposed to 5FU via the modulation of mitochondrial function, ER stress, and the apoptotic signaling pathway. After the treatment of 5FU-resistant SNU-C5 (SNU-C5/5FUR) cells with 5FU, the knockdown of PGC-1α increased the sensitivity of these cells to this anti-cancer drug via a reduction in the mitochondrial function and the induction of ER stress and apoptosis. Several studies have shown that PGC-1α is related to energy metabolism, proliferation, and mitochondrial biogenesis in cells [30,31,32]. In addition, several studies have indicated that PGC-1α contributes to the generation of ROS, the regulation of metastasis, drug resistance, and the proliferation of cancer cells [23,33,34,35]. Our findings confirmed, for the first time, that 5FU-resistant CRC cells express a high level of PGC-1α compared with wild type CRC cells. In breast cancer cells, the expression of PGC-1α induces drug resistance by modulating the bioenergetic capacity of the cancer cells [23]. Moreover, we confirmed that the knockdown of PGC-1α reduced the cell viability after treatment with 5FU, and increased the cell death in 5FU-resistant CRC cells. These results indicate that PGC-1α could play a key role in drug resistance in CRC cells.

PGC-1α is a major transcriptional co-activator that plays important roles in modulating mitochondrial biogenesis, oxidative metabolism, and energy homeostasis in various tissues and pathologic conditions [36,37,38]. With respect to the latter, PGC-1α has been documented to function in a wide array of processes, including glycolysis, oxidative phosphorylation, fatty acid oxidation, glucose-derived lipogenesis, glutamine-derived lipogenesis, and ROS clearance in several types of cancer cells, including melanoma, lung cancer, colon cancer, liver cancer, and breast cancer [39,40,41,42,43]. Interestingly, we found that the mitochondrial mass was not altered after treatment with 5FU in 5FU-resistant CRC cells, whereas the knockdown of PGC-1α significantly reduced the mitochondrial mass. Also, the silencing of PGC-1α decreased the mitochondrial function, as evidenced by the reduction in the mitochondrial complex I and IV activities and the oxygen consumption ratio. The production of ROS during chemotherapy, including 5FU-based therapies, is a critical mechanism by which anti-cancer drugs exert their effects [44]. We confirmed that the downregulation of PGC-1α induced mitochondrial ROS and inhibited the activity of antioxidant enzymes after treatment with 5FU in 5FU-resistant CRC cells. These results indicate that PGC-1α contributes to the maintenance of mitochondrial morphology and oxidative phosphorylation, and protects 5FU-resistant CRC cells from ROS-induced damage.

Production of mitochondrial ROS induces ER stress [45]. Furthermore, expression of PGC-1α regulates ER stress, resulting in ROS-ER stress-mediated apoptosis [46]. Indeed, our previous study indicated that an elevated ROS production induced ER stress, leading to apoptosis in CRC cells [47]. Our results showed that the 5FU-mediated ER stress was partially suppressed in the 5FU-resistant CRC cells by inhibiting the activation of PERK, suggesting that the 5FU-resistant CRC cells might be resistant to 5FU-induced ER stress. Interestingly, in the presence of 5FU, silencing PGC-1α significantly triggered the activation of ER stress markers, such as PERK, IRE1α, ATF4, and CHOP, in the 5FU-resistant CRC cells. A recent study has shown that diabetes-induced ER stress represses PGC-1α through the induction of the CHOP expression [48]. These findings suggest that PGC-1α is closely linked to ER stress, and that it plays a pivotal role in preventing ER stress in drug-resistant CRC cells.

**Figure 6:**
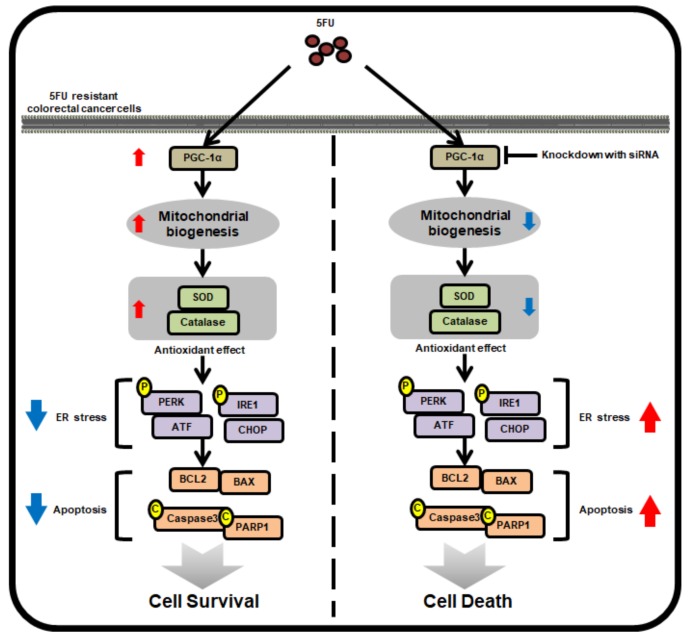
Schematic illustrating the mechanism by which PGC-1α mediates its anti-5FU effects in 5FU-resistant CRC cells. In 5FU-resistant CRC cells, treatment with 5FU promotes the expression of PGC-1α, which induces mitochondrial biogenesis and the activity of antioxidant enzymes, resulting in the augmentation of cell survival by inhibiting 5FU-induced ER stress and apoptosis. However, silencing PGC-1α blocks these protective effects in 5FU-resistant CRC cells against treatment with 5FU, indicating that PCG-1α is associated with 5FU-resistance in CRC cells.

In this study, we show, for the first time, that 5FU-resistant CRC cells express high levels of PGC-1α compared to 5FU-sensitive CRC cells. Increased PGC-1α enhances 5FU resistance in CRC cells by modulating the mitochondrial function, the production of ROS, anti-cancer drug-induced ER stress, and apoptotic pathways (Figure 6). Further study is necessary in order to understand how the expression of PGC-1α is induced in drug-resistant CRC cells, and to investigate whether PGC-1α is involved in the anti-cancer drug resistance of other CRC cells. Taken together, this study indicates that PGC-1α regulates the mitochondrial function and 5FU resistance in 5FU-resistant CRC cells through the inhibition of 5FU-mediated ER stress and apoptosis, suggesting that the targeting of PGC-1α might be a novel therapeutic strategy in combination with 5FU chemotherapy to combat 5FU-resistant CRC cells.

## 4. Materials and Methods

### 4.1. Preparation of 5FU

5FU was obtained from Sigma-Aldrich (St. Louis, MO, USA) and was dissolved in dimethyl sulfoxide (DMSO). This solution was then filter-sterilized through a 0.22 µm pore filter (Sartorius Biotech GmbH, Gottingen, Germany), and the aliquots were stored at 4 °C until use.

### 4.2. Cells and Cell Culture

The human colorectal cancer cell lines (5FU-sensitive SNU-C5 cells; SNU-C5/WT and 5FU-resistant SNU-C5 cells; SNU-C5/5FUR) were obtained from the Chosun University Research Center for Resistant Cells (Gwangju, Republic of Korea). The cells were maintained in RPMI 1640 supplemented with 10% fetal bovine serum, L-glutamine, and antibiotics (Biological Industries, Beit Haemek, Israel) at 37 °C with 5% CO2 in a humidified incubator.

### 4.3. Cell Viability Assay

The exponentially growing colon cancer cells were incubated in 96-well plates with 5FU (140 µM) for 24 h. The cell viability was determined by a modification of the 3-(4,5-dimethylthiazol-2-yl)-2,5-diphenyltetra-zolium bromide (MTT) assay, in which the tetrazolium salt (3-(4,5-dimethylthiazol-2-yl)-5-(3-carboxymethoxy-phenyl)-2-(4-sulfophenyl)-2-tetrazolium) was converted to formazan by mitochondrial dehydrogenase in the viable cells. The formazan was quantified by measuring the absorbance at 570 nm using a microplate reader (BMG Labtech, Ortenberg, Germany).

### 4.4. Quantitative Real-Time PCR (qRT-PCR)

The qRT-PCR analysis was performed using the Maxima SYBR Green/ROX qPCR Master Mix (Thermo Fisher Scientific, Waltham, MA, USA). The qRT-PCR reaction using the StepOnePlus Real-Time PCR system (Thermo Fisher Scientific) was performed under cycling conditions of 95 °C for 45 s (denaturation), 60 °C for 45 s (annealing), and 72 °C for 60 s (extension), for 40 cycles. The gene expression level normalized to β-actin was calculated using the ΔΔCt method with reference to the SNU-C5/5FUR control. The primer sequences were as follows: PGC-1α forward, 5′-CACCAGCCAACACTCAGCTA-3′; PGC-1α reverse, 5′-GTGTGAGGAGGGTCATCGTT-3′; β-actin forward, 5′-AACCGCGAGAAGATGACC- 3′; β-actin reverse, 5′-AGCAGCCGTGGCCATCTC-3′.

### 4.5. Western Blot Analysis

The total cell protein was extracted using a radioimmunoprecipitation assay (RIPA) lysis buffer (Thermo Fisher Scientific, Waltham, MA, USA). The cell lysates were separated using sodium dodecyl sulfate-polyacrylamide gel electrophoresis, and the proteins were transferred to polyvinylidene fluoride membranes (PVDF; Millipore, Billerica, MA, USA). The PVDF membranes were blocked with 5% skim milk and were incubated with primary antibodies against PGC-1α (Santa Cruz Biotechnology, Dallas, TX, USA), phospho-protein kinase R-like endoplasmic reticulum kinase (p-PERK; Santa Cruz Biotechnology), PERK, phospho-inositol-requiring enzyme 1α (p-IRE-1α; Santa Cruz Biotechnology), IRE1α, B-cell lymphoma 2 (BCL2; Santa Cruz Biotechnology), Bcl-2-associated X protein (BAX; Santa Cruz Biotechnology), cleaved caspase-3 (Santa Cruz Biotechnology), cleaved poly [ADP-ribose] polymerase 1 (PARP1; Santa Cruz Biotechnology), activating transcription factor 4 (ATF4; Novus Biologicals, Littleton, CO, USA), CCAAT-enhancer-binding protein homologous protein (CHOP; Novus Biologicals), and β-actin (Santa Cruz Biotechnology). After incubation, the membranes were incubated with peroxidase-conjugated goat anti-mouse or anti-rabbit IgG secondary antibodies (Thermo Fisher Scientific). The protein bands were visualized by using enhanced chemiluminescence reagents (Amersham Biosciences, Uppsala, Sweden).

### 4.6. Inhibition of PGC-1α Expression by Small Interfering RNA (siRNA)

SNU-C5/5FUR cells were seeded in 60 mm dishes and were transfected with siRNA in serum-free Opti-MEM (Gibco BRL) by using Lipofectamine 2000, following the manufacturer’s instructions (Thermo Fisher Scientific). After transfection with siRNA for 48 h, the cells were treated with 5FU for 24 h. The siRNA used to block PGC-1α (The PGC-1α siRNA no. 1 sequence: 5′-UCACCGAGACCGACGUUAA-3′, no. 2 sequence: 5-GAUCGAGCAUGGUCCUCUU-3′, no. 3 sequence: 5′-AGAUGUGUAUCACCCAGUA-3′, and no. 4 sequence: 5′-GACCGUUACUAUCGUGAAA-3′) and a scrambled sequence (The scrambled siRNA no. 1 sequence: 5′-UGGUUUACAUGUCGACUAA-3′, no. 2 sequence: 5′-UGGUUUACAUGUUGUGUGA-3′, no. 3 sequence: 5′-UGGUUUACAUGUUUUCUGA-3′, and no. 4 sequence: 5′-UGGUUUACAUGUUUUCCUA-3′) were purchased from Dharmacon (Lafayette, CO, USA).

### 4.7. Flow Cytometric Analysis

To measure the reactive oxygen species (ROS), the SNU-C5/5FUR cells were stained with dihydroethidium (DHE) (Sigma) for 30 min at 37 °C, and were washed with PBS (phosphate-buffered saline) three times. To determine if the cells were apoptotic, they were stained with annexin V-fluorescein isothiocyanate (FITC) and propidium iodide (PI) (Sigma). Each sample was quantitatively analyzed using a CyFlow Cube 8 (Sysmex Partec, Münster, Germany). A data analysis was performed using the FCS Express software package (De Novo Software).

### 4.8. Immunofluorescence Staining

The SNU-C5/WT and SNU-C5/5FUR cells on cover glass coverslips were treated with 5FU for 24 h. MitoTracker^®^-Red probe (Thermo Fisher Scientific) was directly added into the culture media and incubated for 10 min at 37 °C in the dark. After washing three times in PBS, the cells were fixed in a 4% paraformaldehyde solution for 10 min. The stained slides were imaged using a fluorescent microscope (ZEISS, Oberkochen, Germany).

### 4.9. Measurement of Oxygen Consumption

The SNU-C5/WT and SNU-C5/5FUR cells were seeded in a 96-well culture plate and treated with 5FU and PGC-1α siRNA. The measurements of the oxygen consumption were performed using extracellular oxygen consumption assay kits (Abcam, Cambridge, UK), following the manufacturer’s instructions.

### 4.10. SOD and Catalase Activity

To measure the SOD activity, SNU-C5/WT and SNU-C5/5FUR cells were harvested from the culture plates by scraping with a rubber policeman on ice. The cell proteins were extracted using a RIPA extraction buffer. The cell lysates were reacted with the SOD, and were then measured every minute in a microplate reader (BMG Labtech) at 450 nm for 10 min. To measure the catalase activity, the cell lysates were incubated with 20 mM H^2^O^2^ in 0.1 M Tris-HCI (Sigma-Aldrich) for 30 min, and then 50 mM of Amplex Red reagent (Thermo Fisher Scientific) and 0.2 U/mL of horseradish peroxidase (Sigma-Aldrich) were added and incubated for 30 min at 37 °C. Alterations in the absorbance were quantified by measuring the absorbance at 563 nm using the aforementioned microplate reader.

### 4.11. Mitochondrial Complex I and IV activity

The mitochondrial fractions (0.6 mg/mL) were incubated for 3 min in a mitochondrial extraction buffer (250 mM sucrose, 50 mM potassium-phosphate, 1 mM KCN, 50 μM decylubiquinone, and 0.8 μM antimycin, pH 7.4). The complex I activity was measured using a complex I enzyme activity assay kit (Abcam), following the manufacturer’s instructions. The absorbance was determined from the rate of the oxidation of NADH (100 mM) at 450 nm for up to 30 min. The complex IV activity was measured using a complex IV enzyme activity kit (Abcam), and adding cell lysates to 75 μL of cytochrome c previously reduced with sodium borohydride, and then determining the absorbance at 550 nm.

### 4.12. Statistical Analyses

The results are expressed as the mean ± standard error of the mean (SEM). All of the experiments were analyzed by ANOVA. In some experiments, this was followed by a comparison of the treatment mean with the control using a Bonferroni–Dunn test. The data were considered significantly different at values of *p* < 0.05.

## 5. Conclusions

In this study, we investigated whether the expression of PGC-1α increased in the 5FU-resistant CRC cells compared with the wild type CRC cells, and enhanced 5FU resistance via the modulation of the mitochondrial function, production of ROS, anti-cancer drug-mediated ER stress, and apoptosis. The knockdown of PGC-1α increased the production of ROS and induced an apoptotic pathway through the 5FU-mediated ER stress. The results indicated that the regulation of PGC-1α may be the critical factor for cancer therapy in 5FU-resistant CRC cells.

## Supplementary Materials

The following are available online at https://www.mdpi.com/1422-0067/20/7/1707/s1. Figure S1. Effects of peroxisome proliferator-activated receptor gamma coactivator 1-alpha (PGC-1α)-siRNA on modulation of the mitochondrial complex; Figure S2. Effects of PGC-1α-siRNA on modulation of catalase and superoxide dismutase (SOD) activity; Figure S3. Effect of PGC-1α-siRNA in SNU-C5/5FUR cells on endoplasmic reticulum (ER) stress; Figure S4. Effect of PGC-1α-siRNA in SNU-C5/5FUR cells on apoptosis.
