# Supplementary material for: PGC-1α Controls Mitochondrial Biogenesis in Drug-Resistant Colorectal Cancer Cells by Regulating Endoplasmic Reticulum Stress
Source: Int J Mol Sci. 2019 Apr 5;20(7):1707. doi: 10.3390/ijms20071707 (PMC6480203; doi:10.3390/ijms20071707)
Supplement: Supplementary file 1 [file ijms-20-01707-s001.zip › 19.04.03_ijms-478652-supplementary.docx]

**Supplemental Figures**


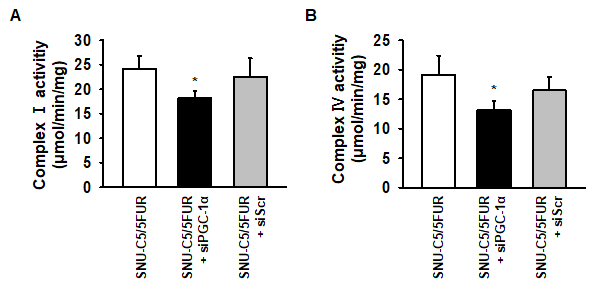


**Supplemental Figure S1**. Effects of peroxisome proliferator-activated receptor gamma coactivator 1-alpha (PGC-1α)-siRNA on modulation of the mitochondrial complex. (A) Mitochondrial complex I and (B) IV activity were measured using a microplate reader (n = 3; biological replicates). Values represent means ± SEM; *p < 0.05 vs. control.


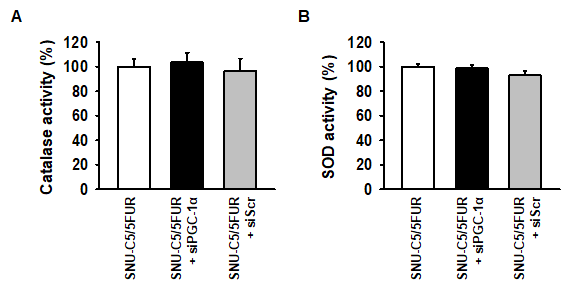


**Supplemental Figure S2**. Effects of PGC-1α-siRNA on modulation of catalase and superoxide dismutase (SOD) activity. (A) Catalase and (B) SOD activity were analyzed using a microplate reader following siPGC-1α transfection (n = 3; biological replicates).


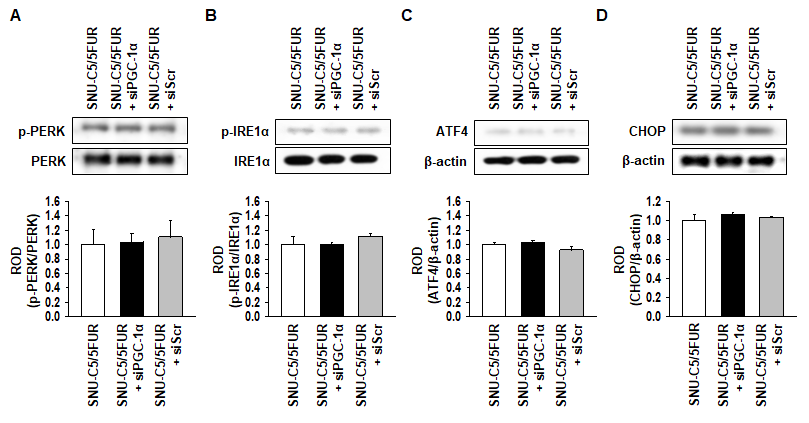


**Supplemental Figure S3**. Effect of PGC-1α-siRNA in SNU-C5/5FUR cells on endoplasmic reticulum (ER) stress. (A–D) The expression levels of ER stress markers, phospho-protein kinase-like endoplasmic reticulum kinase (p-PERK) (A), phospho-inositol-requiring enzyme 1 alpha (p-IRE1α) (B), activating transcription factor 4 (ATF4) (C), and C/EBP homologous protein (CHOP) (D), in siPGC-1α-transfected SNU-C5/5FUR cells (n = 3; biological replicates).


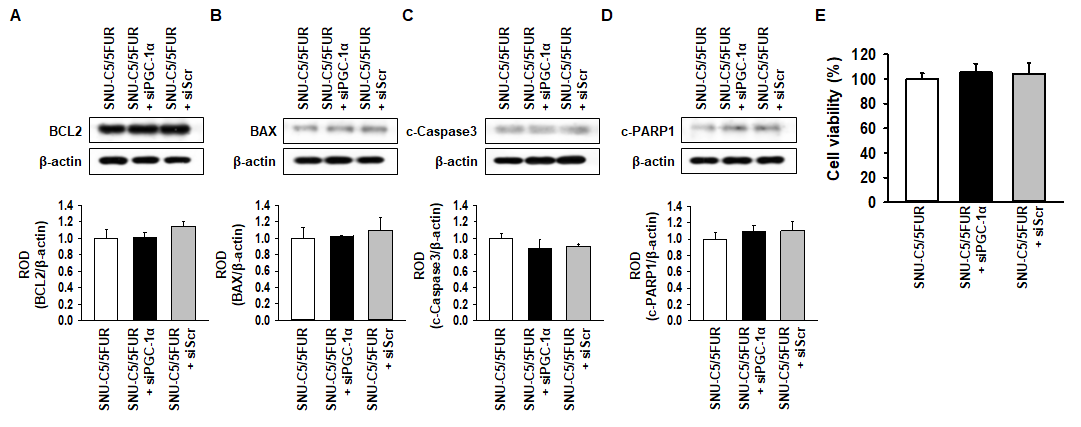


**Supplemental Figure S4**. Effect of PGC-1α-siRNA in SNU-C5/5FUR cells on apoptosis. (A–D) The levels of apoptosis-related proteins, including anti-apoptotic protein B-cell lymphoma 2 (BCL2) (A), and pro-apoptotic proteins Bcl-2-associated X protein (BAX) (B) and cleaved caspase 3 (c-Caspase3; C), and apoptotic protein cleaved Poly [ADP-ribose] polymerase 1 (c-PARP1; D) in siPGC-1α-transfected SNU-C5/5FUR cells. (E) Cell viability of SNU-C5/5FUR transfected with siPGC-1α (n = 3; biological replicates).
