# Supplementary figures and images for: PGC-1α Controls Mitochondrial Biogenesis in Drug-Resistant Colorectal Cancer Cells by Regulating Endoplasmic Reticulum Stress
Source: Int J Mol Sci. 2019 Apr 5;20(7):1707. doi: 10.3390/ijms20071707 (PMC6480203; doi:10.3390/ijms20071707)

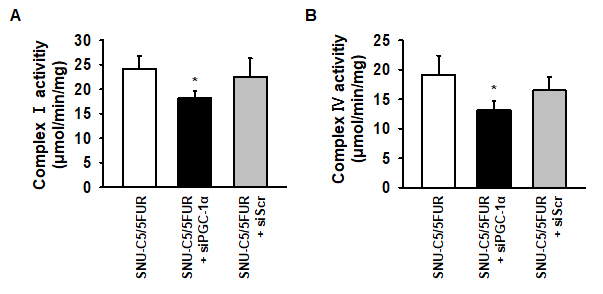

Supplement: Supplementary file 1 [file ijms-20-01707-s001.zip › supplementary figure/S1.tif]

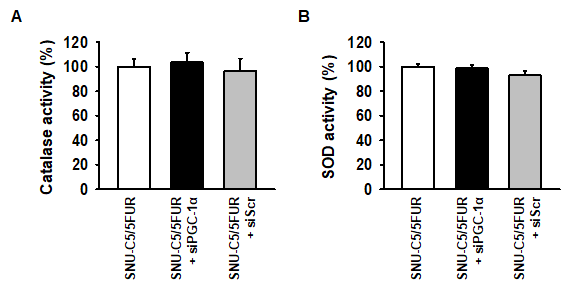

Supplement: Supplementary file 1 [file ijms-20-01707-s001.zip › supplementary figure/S2.tif]

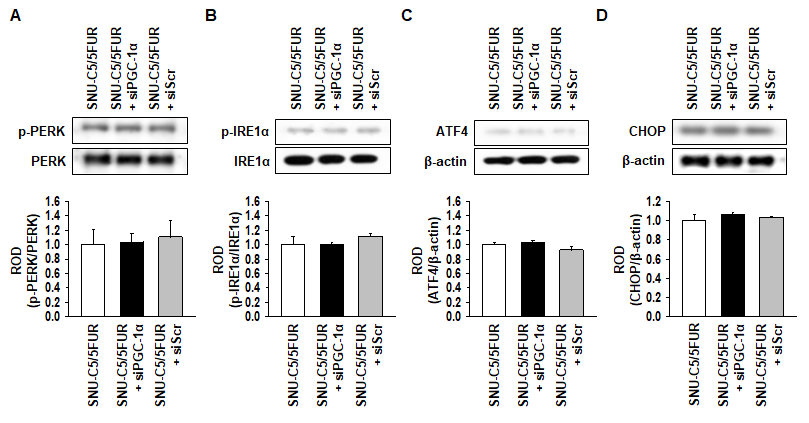

Supplement: Supplementary file 1 [file ijms-20-01707-s001.zip › supplementary figure/S3.tif]

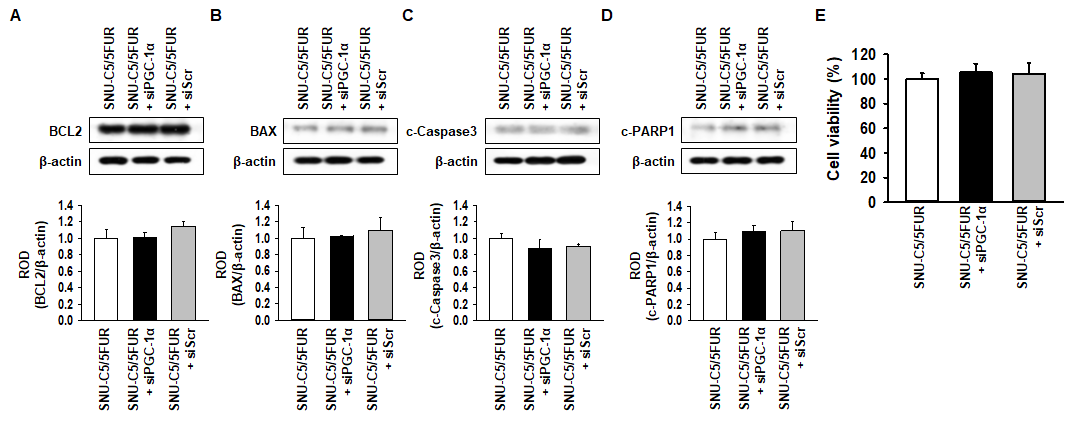

Supplement: Supplementary file 1 [file ijms-20-01707-s001.zip › supplementary figure/S4.tif]
